# Supplementary material for: Are carbon emissions trading and green financial instruments synergistic? -Comprehensive quantitative research based on content analysis
Source: PLoS One. 2024 Mar 7;19(3):e0298601. doi: 10.1371/journal.pone.0298601 (PMC10919601; doi:10.1371/journal.pone.0298601)
Supplement: S1 Data — (ZIP) [file pone.0298601.s001.zip › Data/地级市Z_F工作报告.zip!/╦─┤¿/─┌╜¡╩╨/└┤╫╘╣1⁄2╓┌║┼í╛┬φ┐╦╩2╛▌═°í┐_2017.docx]

各位代表：

　　我代表市人民政府，向大会报告工作，请予审查，并请市政协委员提出意见。

一、2016年工作回顾

　　2016年，面对错综复杂的宏观经济形势，在省委、省政府和市委的坚强领导下，在市人大、市政协的监督支持下，我们紧紧依靠全市人民，勤勉履职、苦干实干，推动了经济社会平稳健康发展。全市地区生产总值1297.7亿元，增长7.8%；地方一般公共预算收入53.7亿元，同口径增长11.4%；农村居民人均可支配收入12491元，增长9.3%；城镇居民人均可支配收入27986元，增长8.5%，实现了“十三五”良好开局。

　　（一）坚持创新发展，发展质量进一步提升

　　发展动能加速培育。落实省委、省政府“项目年”部署，举行重大项目集中开工7次，新开工项目283个；强化项目督查，46个项目如期竣工；实施PPP项目8个，投资总额44.2亿元；235个市级重点项目、11个省级重点项目完成年度投资计划，全社会固定资产投资完成905.5亿元，增长12.7%。积极扩大内需，新增限额以上商贸流通企业34户，实现社会消费品零售总额460.5亿元，增长12.7%。加强“三外”工作，实现对外工程承包营业额4344万美元，实际利用外资4724.8万美元，外贸进出口总额达1.3亿美元。

　　转型升级步伐加快。推进供给侧结构性改革，120万吨钢铁去产能任务提前完成，16户落后产能企业被淘汰，单位GDP能耗下降4.8%；消化房地产库存433万平方米，商品住房去化周期缩短至12.7个月。启动先进制造强市“三百行动”，完成技改投资250.8亿元，新增规模以上企业31户，全市规模工业增加值增长9.4%。大力发展电子商务，实现交易额418.3亿元，居全省第三位。培育发展会展业，举办重点展会22场次，促成交易额9.6亿元。大力发展现代旅游业，新增国家AAAA级旅游景区2个，实现旅游总收入216亿元，增长26.7%。加快发展金融业，新引进域外金融机构3家；各项存款余额、贷款余额分别达1370.3亿元、732.2亿元，比年初分别增加165.8亿元、43.4亿元。新发展现代农业产业示范片5.3万亩，新增种养大户251个、家庭农场128个、农民合作社51个、龙头企业10户，新获批绿色食品2个、无公害农产品2个，农业增加值增长4.0%。

　　创新驱动深入实施。内江国家高新区已获国务院批准。累计建成孵化载体22个、孵化面积13.2万平方米，新建市级以上工程技术中心7个。实施市级以上重大科技成果转化项目41个，新获批国家高新技术企业11户，新增各类科技型小微企业50户。新培育市级以上高新技术产品23个、战略性新兴产品24个。高新技术和战略性新兴产业产值占规模工业总产值比重分别提高3.9、3.8个百分点。

　　（二）坚持协调发展，发展效能进一步增强

　　“双百城市”有序推进。城市总规完成修编，获得省政府批复。实施道路桥梁项目25个、公建配套项目21个，新城核心区“四横七纵”路网骨架全面建成，中心城区建成区面积约80平方公里、常住人口约80万人。改造棚户区危旧房8161套，货币化安置率达82.2%，继续走在全省前列。改造老旧院落116个。拆除违章搭建31万平方米。深化城区交通治堵，桐梓坝大桥南成渝铁路桥拓宽工程竣工，沱江大桥加宽改造按期推进。“城市变景区”进程加快，大洲广场、松山公园改造工程和小青龙河绿道建设进展顺利，塔山复合绿道建成投用。加强城市管理，推进文明城市创建，启动中心城区“交通安全•文明礼让”行动。

　　基础设施逐步改善。积极构建“一带一轴一区”重要交通枢纽，内江城市过境高速公路、川南城际铁路开工，花园滩大桥主体工程完工，新（改）建农村公路1002.9公里，完成渡改桥9座，实现交通投资28.8亿元。联合水库、两河口水库建设加快，长桥水库如期开工。

　　县域经济不断壮大。各县（区）竞相发展势头强劲，底部基础进一步夯实。市中区用足老工业基地调整改造政策，大力发展高新技术和战略性新兴产业，改造提升传统商贸业。东兴区抢抓“高铁时代”新机遇，大力发展电子商务、现代物流和会展业。资中县积极发挥国家农业科技园区核心区作用，大力发展现代农业。隆昌县主动融入川渝合作，大力发展先进制造业和现代旅游业。威远县加快工业转型升级，大力发展新材料产业和生态旅游。内江经开区推进产城融合发展，国家级经开区示范引领作用更加明显。启动县城开发边界划定工作，县城控规覆盖率均超过95%，一大批公共配套设施建成投用。17个国、省重点镇加快建设，建成幸福美丽新村300个。

　　（三）坚持绿色发展，发展环境进一步改善

　　沱江流域综合治理全面启动。将内江沱江流域综合治理和绿色生态系统建设与保护作为“世代工程”，启动编制《内江市沱江流域综合治理和绿色生态系统建设与保护规划》，加快沱江内江段、威远河、隆昌河、大清流河等主要流域治理，城区20个涉水项目有序推进，城市集中式饮用水源水质达标率100%。

　　生态环境保护切实加强。打响污染防治“三大战役”，制定《推进绿色发展建设美丽内江的实施方案》，投入资金11.5亿元，实施环保项目107个，竣工项目56个。中心城区PM10、PM2.5年均浓度分别下降8.1%、11.6%。启动大规模绿化内江行动，森林覆盖率达33.76%，我市被评为“四川绿色发展范例城市”。立案查处环境行政处罚案件92件，立案查处率100%。环保突出问题整改通过环保部西南督查中心督查验收，内江做法在西南各省推广。

　　（四）坚持开放发展，发展活力进一步释放

　　招商引资成效明显。借力“中外知名企业四川行”、第十六届西博会等重大平台，签约项目82个。成功举办第二届川南电商博览会、第七届大千龙舟经贸文化节、第九届中国国际中小企业交易会等活动，内江对外知名度得到提升。大力实施返乡创业“回家工程”，新增返乡创业人员3802人，创办企业473户，总投资142.4亿元。全力推动签约项目落地，新签且履约项目453个。全市到位市外资金增长12.8%。

　　开放合作不断深化。参与编制川南经济区“十三五”发展规划，积极推进川南一卡通、川南统一电话区号等合作事项。签署川渝合作荣隆工业园框架协议。深化市校合作，与北京大学、北京航空航天大学、西北工业大学等高校达成合作意向，浙江大学、重庆大学技术转移中心挂牌运营。

　　重点改革扎实推进。实施8项国家级、17项省级改革试点，54项年度改革任务顺利完成。农村土地承包经营权确权登记通过省级验收，市中区农村土地退出“三换”模式入选中国改革（2016）年度案例。“放管服”改革全面深化，免收行政事业性收费18项，取消前置审批条件55个，压缩审批环节近100个，审批办件提速90%以上，在县级层面试点建立行政审批局，政务环境进一步改善。推动商事制度改革，新发展市场主体1.6万户。深化财税体制改革，“营改增”全面推开。推进投融资体制改革，加快组建城市发展基金、产业发展基金。隆昌农商行顺利挂牌，内江农商行获批筹建。公车改革全面完成，公共资源交易、国资国企、科技等领域改革取得积极进展。

　　（五）坚持共享发展，发展成果进一步惠民

　　脱贫攻坚首战告捷。大力实施精准脱贫，投入资金12亿元，实现63个贫困村退出、2.44万人脱贫。有序实施易地扶贫搬迁，安置贫困人口1794人。全面启动“四好村”创建，建成省级、市级“四好村”32个、169个。创新扶贫机制，建立“五项基金”，“两集中、三权六化”扶贫模式获得国务院扶贫办肯定。

　　民生实事全面完成。民生支出140.9亿元，占一般公共预算支出的70.4%，比全省平均水平高5.4个百分点，“十项民生工程”“20件民生大事”全面完成。新增就业4.9万人。五大保险参保覆盖人数增加6.7万人，新农合居民参合率达99.8%，统一城乡基本医疗保险工作全面启动。建成全国首批“12349”居家养老服务平台，城乡居家和社区养老服务覆盖率分别达73.3%、35.1%。改造农村危房3705户。完成农村水井水质监测2485口。启动农村饮水安全提升工程，新增农村供水受益者5万人。

　　社会事业继续加强。惠民工程加快建设，市文化馆完成主体工程，内江师院整体迁建项目顺利签约。新（改、扩）建公办幼儿园15所，完成义务教育学校标准化建设60所，高考万人本科上线率保持全省前列，藏区“9+3”中职免费教育毕业生100%就业，2.2万名家庭经济困难寄宿制学生获得生活补助。全面取消城市公立医院药品加成，成功消除以市为单位的疟疾，“全面两孩”政策得到落实。新建农村广播影视公共服务网点83个，开展群众文化活动700余场次，公共文化设施全部免费开放。承办省级以上大型体育赛事10场次，开展“周末快乐体育” 活动500余场次。

　　和谐局面持续巩固。坚决守住“三条底线”，加强重点行业领域安全生产监管，没有发生较大及以上事故，安全生产形势总体平稳。加强食品药品监管，没有发生重大食品药品安全事件。创新社会治理，积极开展临时救助，扎实推进“法律七进”活动，完善多元化矛盾化解机制。坚持领导接访、下访和包案制，畅通群众诉求表达渠道。加强派出所、司法所规范化建设，依法打击违法犯罪活动，社区矫正和刑满释放人员安置帮教工作扎实推进，公众安全感满意率达95.6%。

　　（六）加强自身建设，行政能力进一步提高

　　深入开展“两学一做”学习教育，持之以恒落实中央八项规定精神和省、市十项规定，“三公经费”同比下降20.4%。自觉接受人大法律监督、工作监督和政协民主监督，办理人大代表议案3件、建议90件，办理政协提案252件。广泛听取民主党派、工商联、无党派人士、人民团体的意见。积极支持工会、共青团、妇联、残联等群团组织开展工作。大力推进依法行政，坚持政府常务会会前学法，完善法律顾问制度，全面实施重大行政决策合法性审查。规范行政处罚裁量权，强化行政复议工作。建成综合性市级政务服务平台，实施“三张清单”制度，推行“双随机、一公开”监管，全市行权平台办件47万余件，办件量居全省第三位。大力整治侵害群众利益的不正之风和腐败问题，政纪处分184人。

　　国防教育、国防动员、人民防空、双拥共建、优抚安置等工作取得新进步。机构编制、统计调查、气象、水文、防灾减灾、检验检疫、无线电管理、保密、档案、史志、外事侨务、民族宗教等工作实现新发展。新闻出版、红十字、慈善、老龄、妇女儿童等事业取得新成绩。

　　各位代表，过去的一年，取得这样的成绩，是省委、省政府坚强领导、深切关怀的结果，是市委统揽全局、正确决策的结果，是市人大、市政协加强监督、大力支持的结果，是全市广大干部群众勤奋工作、埋头苦干的结果。在此，我代表市人民政府，向420万内江人民，向人大代表、政协委员、各民主党派、工商联、无党派人士、各人民团体，向驻内部队、武警官兵、政法干警和民兵预备役人员，向关心、支持和参与内江建设发展的各界朋友，表示衷心的感谢！

　　各位代表，我们也清醒地认识到，内江经济社会发展还存在不少问题和挑战，地区生产总值增速、服务业增加值增速、建筑业增加值增速等指标未能实现预期目标。经济总量偏小、产业结构不优，实体经济发展仍然困难。投资后劲不足，大项目、好项目少，产业项目缺乏，资源要素日益趋紧。财政占经济总量比重偏低，税源培植差距较大。农村基础设施薄弱，农民增收门路较窄，精准脱贫任务艰巨。城市综合配套不够完善，城区交通拥堵还没有明显缓解。教育、医疗、环境保护、饮水安全等还有不少问题需要解决。少数政府部门和公务人员担当意识不够、办事效率较低。对此，我们将以创新的精神、务实的作风、统筹的办法，认真加以改进。

二、2017年主要目标任务

　　今年是实施“十三五”规划的重要一年，是推进供给侧结构性改革的深化之年，做好各项工作至关重要。今年政府工作的总体要求是：全面贯彻党的十八大和十八届三中、四中、五中、六中全会精神，认真落实以习近平同志为核心的党中央治国理政新理念新思想新战略，围绕“五位一体”总体布局和“四个全面”战略布局，贯彻落实省委十届八次、九次全会精神，按照市第七次党代会、市委七届二次全会、市委经济工作会议部署，坚持稳中求进工作总基调，坚持以提高发展质量和效益为中心，坚持以推进供给侧结构性改革为主线，紧扣“两个率先”“三个基本建成”奋斗目标，致力于科学发展、加快发展、专注发展、转型发展，深入实施“三大发展战略”，突出全面创新改革“一号工程”，突出内江沱江流域综合治理和绿色生态系统建设与保护“世代工程”，突出脱贫攻坚“头等大事”，全面做好稳增长、促改革、调结构、惠民生、防风险各项工作，打造“内江实践方案”升级版，加快建设幸福美丽内江。

　　今年全市经济社会发展的主要预期目标是：地区生产总值增长7.8%，其中，第一产业增加值增长3.6%、第二产业增加值增长8.5%（其中，规模工业增加值增长9.0%、建筑业增加值增长12.5%）、第三产业增加值增长9.0%；全社会固定资产投资增长10.5%；社会消费品零售总额增长12.0%；地方一般公共预算收入同口径增长7.8%；城乡居民人均可支配收入分别增长8.5%、9.0%；居民消费价格指数涨幅控制在3.0%以内；城镇登记失业率控制在4.5%以内；完成省下达的节能减排和环境保护目标任务。

　　这些目标，充分考虑了与全国全省同步全面小康、稳定市场信心和增加就业的需要，为稳增长、调结构、转方式预留了一定空间；充分考虑了内江发展不足、发展质量不高的实际，对接了内江经济社会发展“十三五”规划的安排；充分考虑了项目投资、产业发展等方面的支撑条件和生态环境的承载能力，符合稳中求进工作总基调。

　　三、2017年重点工作

　　围绕总体要求和预期目标，我们将重点抓好以下工作：

　　（一）努力稳定经济增长

　　持续扩大有效投资。积极抓好“项目年”工作，创新项目生成、评审提升、综合推进机制，抓好28个省级重点项目、287个市级重点项目建设，完成省级重点项目投资38.6亿元、市级重点项目投资215亿元，完成全社会固定资产投资1000亿元以上。开工建设高新区白马新材料产业园，加快建设恒通动物制剂生产基地、隆昌年产1500台起重设备等项目，竣工投产厦门新能源新材料及电子产业工业园、金镭铸业年产3万吨专用锻铸件等项目，完成工业投资230亿元。引导社会资本投向民生领域，实现民生及社会事业投资152.5亿元。

　　深入推进新型城镇化。促进农业转移人口有序市民化，全市城镇化率提高1.5个百分点。强化规划引领，加强城市总规实施。扩大规模，加速推进“一中心四组团”建设，通过邱家嘴立交桥南延线、黄荆坝大桥、汉渝大道上跨内宜高速桥等项目连接各组团，新开工“双百城市”建设项目132个，完成年度投资31亿元，力争中心城区建成区面积达85平方公里、常住人口达85万人。完善设施，深化“城市基础设施建设年行动”，推进地下管廊和海绵城市建设，加快北环线I、II段和玉屏街、东城路等道路建设，开工建设太白路北延线林荫大道。打造环境，实施城市变景区项目11个，启动建设清溪湿地北段公园、长江森林公园、千水湖公园、五星水库主题公园等旅游景观项目；持续推进旧城改造，改造棚户区危旧房7083套，改造老旧院落87个；打好城区交通治堵“五年持久战”，实施项目14个，完成西林大桥南北节点改造，加快东兴大道南延线、春江路等项目建设，启动沱中路、蟠龙路北段等项目改造；依托数字化城市管理信息系统，实施精细化管理。因地制宜发展县城新区，做好城市设计，完善综合配套，注重产业培育，建设宜居宜业中等城市。持续抓好17个国、省重点镇建设，发展一批工业、商贸、旅游、文化、农业示范镇。

　　努力激活城乡消费。优化特色街区布局，推进重大商贸设施建设，加快打造谢家河、高铁站片区新兴商圈。做强一批名特优产品专卖店，做大一批特色餐饮品牌，扩大内江消费辐射力。积极培育汽车、信息、文化、会展等消费热点，引导企业开展各类交易会、节庆消费活动。加强产品质量监管，严厉打击假冒伪劣产品，创建放心舒心消费城市。

　　积极提振外贸出口。加快内江海关建设，力争早日建成投用。完善支持政策，鼓励企业走出去，进出口总额超过1.3亿美元。支持企业利用电子商务开展跨境贸易，促进高技术含量和高附加值产品出口。支持外贸新业态加快成长，培育发展服务贸易和服务外包。

　　（二）构建现代产业体系

　　加快工业提质增效。大力实施先进制造强市“三百行动”，发展先进制造企业30户，技改传统制造企业30户，培育制造业名优品牌30个。提升壮大冶金建材、机械汽配、医药化工、食品饮料、电力能源五大传统特色产业，实现产值1750亿元。加快发展高端装备制造、生物医药、新材料等产业，高新技术和战略性新兴产业产值占规模工业总产值的比重分别达31%和22.8%。大力发展信息安全、节能环保等产业，高端成长型产业产值超过100亿元。加大企业培育力度，新增规模以上工业企业20户、5亿元以上企业5户、10亿元以上企业2户，支持更多企业上市。继续落实“工业强市八条措施”，稳定大企业大集团生产经营，帮助停产半停产企业恢复生产。提升七大重点工业园区承载能力，设立工业园区产业发展引导资金，新入驻投资5000万元以上企业（项目）不少于22个，实现主营业务收入1900亿元以上，产业集中度达73%。

　　加快服务业提档升级。优先发展电子商务、现代物流、健康养老等新兴先导型服务业，加快发展研发设计、检验检测、咨询等生产性服务业，推动居民生活服务业转型升级。实施服务业品牌培育“552”工程，集中推进50个重点项目，帮扶50户重点企业，培育20个重点品牌。争创国家电子商务示范城市，建成1个省级电子商务示范基地，电子商务交易额达500亿元。深入实施旅游发展“一三八”计划，推进威远穹窿旅游一期等项目建设，创建国家AAAA级景区1个，旅游总收入超过260亿元。大力发展农产品物流和冷链物流，开工建设川南快递物流园，加快建设苏宁川南管理总部基地及配送中心，吸引更多知名物流企业入驻内江。大力发展金融业，努力拓宽融资渠道，引进域外金融机构2家，提升金融服务实体经济水平。

　　加快农业提速转型。实施“12345”现代农业产业提升行动，培育“甜城味•大千故里优质农产品”品牌，做强国家农业科技园区和威远镇西食品加工园区，打造30万亩稻田综合种养、年产100万头内江黑猪、百里休闲农业三条产业带，建设优质粮油、精品农业、循环农业、创意体验农业四大功能示范区，建成柑橘、果叶桑、血橙、木本油料、无花果五个万亩产业示范片，增加绿色优质农产品供给，打造成渝特大城市的“菜篮子”“米袋子”和“果盘子”。推进现代农业示范县和现代林业、畜牧业重点县建设，新建高标准农田19万亩，严格保护耕地，稳定粮食产量。加快培育新型农业经营主体，培育种养大户200个、家庭农场50个、农民合作社20个、龙头企业5户。建设幸福美丽新村150个，省级、市级“四好村”分别达15%、20%。推进“互联网+农业”，积极开发农业多种功能，大力发展休闲农业、乡村旅游等新产业新业态，加强农民职业技能培训，持续增加农民收入。统筹城乡发展，推动基础设施和公共服务向农村延伸。

　　（三）推进全面改革创新

　　深化供给侧结构性改革。持续推进“三去一降一补”，淘汰煤炭落后产能9万吨以上，有序稳妥处置“僵尸企业”。加大棚改货币化安置力度，着力消化商品房库存。支持企业市场化、法治化债转股，防范地方融资平台债务风险。落实富余电、直购电政策，推动天然气转供变直供，加大减税降费力度，降低实体经济生产经营成本。抓好“一提一创一培”，实施质量提升工程，打造一批示范项目；开展品牌创建行动，鼓励企业争创中国质量奖、四川质量奖，新增驰名商标1件、著名商标4件；弘扬“工匠精神”，培育“百年老店”，增强内江产品竞争力。

　　推进重点领域改革。深化农村产权制度改革，抓好全国第二批农村改革试验区、全省农村改革综合试验区建设。实施公务员职务与职级并行、生育保险与基本医疗保险合并两项全国改革试点。深化国资国企改革，完善现代企业制度。深化财税体制改革，转变财政支持产业发展方式。深化商事制度改革，新发展市场主体1.3万户。深化金融体制改革，完成内江农商行组建。同步推进教育、卫生、科技、公共资源交易、事业单位分类等重点领域改革，支持国防和军队改革。

　　抓好创新驱动发展。提升重点园区创新能力，加快返乡创业园、大学生创新创业园建设，构建一批众创空间。建设一批重点实验室、工程实验室、院士（专家）工作站，新建市级以上工程技术中心5个。实施市级以上重大科技成果转化项目25项，新认定市级以上高新技术企业10户。深化市校合作，鼓励企业、科研院所、高校开展重大关键技术联合攻关。探索军民融合深度发展新模式，促进“军转民”“民参军”双向转化。继续实施人才发展“三百计划”，培养一批企业精英、创新人才和创业先锋。

　　（四）提升开放型经济水平

　　充分利用开放平台。打造引进来的强磁场，积极参加“第二届中国西部（四川）国际投资大会”“中外知名企业四川行”等省级平台活动，办好第三届川南电商博览会、第八届大千龙舟经贸文化节等活动，加快引进一批优质项目，确保到位市外资金增长10.5%。深入实施返乡创业“回家工程”，新增返乡创业人员1000人、返乡创业企业100户，完成投资30亿元。

　　积极融入区域合作。融入国家“一带一路”和长江经济带发展战略，抢抓国家全面实施《成渝经济区区域规划》和《成渝城市群发展规划》等重大机遇，打造成渝经济区投资洼地。融入川南经济区一体化发展，落实《川南轨道交通一体化合作协议》等专项协议，推动产业发展互补互促、基础设施互联互通、民生工程互惠互利。

　　努力增强招商实效。以世界500强、中国500强、民企500强为主攻方向，开展平台招商、以企招商、精准招商，力争在产业项目引进上实现突破。及时研究出台配套措施，着力破解招商瓶颈；做好签约项目跟踪服务，确保省政府督办项目履约率、开工率、资金到位率分别超过90%、80%、30%。

　　（五）完善城乡基础设施

　　加强重要交通枢纽建设。启动“一带一轴一区”重要交通枢纽三年推进行动，完成交通投资20亿元以上。推动川南城际铁路、成都经新机场经威远至自贡铁路、绵遂内城际铁路等项目建设。加快内江城市过境高速公路建设，开工建设成都经威远至宜宾高速公路，做好南溪至内江高速公路前期工作，规划论证井研至资中至乐至高速公路。抓好东泸路、资荣路、内威路、资东路等快速通道前期工作，力争开工建设资威路快速通道。改造提升国省道和县乡道，完善农村路网。启动建设渡改桥10座。加快枢纽站场建设，推进内江综合客运中心站、内江公交枢纽中心站等项目建设，提高客运、货运周转流动性、通畅性。

　　加强水利基础设施建设。盯住全市人民关心的饮水安全问题，开工建设向家坝灌区北总干渠一期向内江引水工程。积极参与长征渠引水工程规划修编，大力实施大中型灌区续建配套与节水改造，加快联合水库、资中县河库联网输水工程等项目建设，实施“五小水利”工程，完成水利投资6亿元。

　　加强其它基础设施建设。构建“信息高速公路”，持续抓好 “宽带中国”示范城市和“宽带乡村”试点工程，力争在全省率先实现行政村宽带网络全覆盖。深入推进宽带网络大提速，户均宽带同比提速50%。完善内江大数据中心功能，创建信息惠民国家示范城市。加大中低压配电网改造力度，完成74个村电网改扩建。配合抓好页岩气综合开发，促进就地转化升值。

　　（六）建设美丽四川先行区

　　突出沱江流域综合治理。完成《内江市沱江流域综合治理和绿色生态系统建设与保护2016—2020年重大项目规划》，编制内江沱江流域项目投资负面清单，建设内江沱江流域信息管理平台，设立内江沱江流域综合治理专项基金，打造沱江流域水生态环境治理典范城市。加快推进黄河镇水库环境综合整治、谢家河水环境整治工程（一期）等项目建设。

　　打好污染防治“三大战役”。实施大气污染防治“五大工程”，加快重点行业污染治理，淘汰黄标车2500辆，取缔燃煤锅炉11个，确保中心城区PM2.5年均浓度比2015年下降10%。实施水污染防治“四大工程”，编制实施沱江内江段及球溪河、威远河、隆昌河“1+3”水体达标方案；全面推行河长制，综合治理市域内8条主要河流；完成城区饮用水取水口上移工程，推进内江三水厂、第二污水处理厂等涉水项目建设，开展11条城市黑臭水体整治；加快县城、园区、乡镇污水处理设施建设，乡镇集中式饮用水源水质达标率提高6个百分点。实施土壤污染防治“三大工程”，污染地块安全利用率等指标完成省下达任务。

　　促进绿色低碳循环发展。推进工业废气、废水、废物的综合治理和回收再利用，开展企业清洁生产和重点产业园区循环化改造。加强重点领域节能减排，单位工业增加值能耗下降4%以上。支持企业推行绿色设计、开发绿色产品、建设绿色工厂、打造绿色供应链。鼓励绿色消费、绿色出行、绿色居住，建成绿色建筑150万平方米。

　　加强生态建设与保护。深入实施大规模绿化内江行动，深化天然林保护和新一轮退耕还林、水土流失治理，巩固退耕还林成果31.7万亩。推进“八大绿化行动”，实施人工造林3.2万亩，其中，完成沱江干流绿化1万亩。加快创建省级森林城市，支持三县创建省级绿化模范县，全市森林覆盖率达34%。深入实施城乡环境综合整治，开工建设内江市垃圾处理中心，完善县城、乡镇垃圾处理设施。加强环境监管执法，严厉打击环境违法行为。

　　（七）全力保障改善民生

　　打好脱贫攻坚战。深入开展“五个一批”脱贫攻坚行动，大力实施住房安全、产业发展、低保兜底、医疗救助等“22个扶贫专项计划”，确保85个贫困村退出、27779人脱贫。推进易地扶贫搬迁，改善贫困群众生产生活条件。落实“十免四补助”，开展贫困人口免费健康体检，全额报销贫困人口在县域内属于政策范围的住院费用。继续实施“雨露计划”“栋梁工程”，确保义务教育阶段贫困学生无1人因贫辍学。大力实施“农村家庭能人”培养计划，拓宽贫困群众就业渠道，确保有劳动能力的贫困家庭每户至少有1人就业。推行“投资收益”扶贫、“菜单式”驻村帮扶、“万企帮万村”等方式，引导社会力量参与脱贫攻坚。

　　扎实开展民生工程。继续实施“十项民生工程”“20件民生实事”，确保民生支出占一般公共预算支出比重不低于全省平均水平。开展“甜城双创”活动，新增就业3.7万人。为2万名家庭经济困难寄宿制学生发放生活补助，为3.8万名农村义务教育在校学生提供免费营养午餐。为65岁以上老人提供免费健康体检。新增养老机构床位4350张，新建城乡社区日间照料中心87个，为11.8万名困难家庭失能老人和80周岁以上高龄老人提供居家养老服务。适时提高低保、特困人员供养和困难残疾人生活补贴标准。改造农村危房2699户。开展1715口农村水井水质监测工作，加快整治水质不合格水井。新增农村供水受益者3.2万人。

　　切实办好社会事业。扎实推进“十大社会事业惠民工程”，启动四川卫生与健康职业学院建设，开工建设市体育中心，加快建设内江师院新校区、市文化中心，确保内江六中高新校区主体竣工，建成市第一人民医院新区（一期）、市中医医院新区（二期）。新（改、扩）建公办幼儿园15所，建设义务教育标准化学校40所，实现东兴区、资中县县域内义务教育基本均衡，巩固高中教育质量，加快发展职业教育，推动高等教育改革发展。加快市第二社会福利院整体迁建和市精神病康复院建设，提升基层医疗服务能力，确保基层医疗卫生机构规范化建设达70%。坚持计划生育基本国策，继续落实“全面两孩”政策。实施公共文化设施提升工程，深入开展“百姓大舞台、公益大讲堂、书画大展场”等文化惠民活动，推进“书香甜城”“高清内江•智慧广电”建设，加强文化遗产保护传承。扎实开展“践行十爱•德耀甜城”主题活动，深化“交通安全•文明礼让”行动，强力推进国家卫生城市、省级文明城市创建。积极备战省第十三届运动会，深化“周末快乐体育”活动。扎实做好机构编制、统计调查、民族宗教、外事侨务、对台工作、友协、保密、档案、史志和无线电管理等工作，大力发展妇女儿童、老龄、红十字和慈善等事业。

　　全力维护安全稳定。严格落实“党政同责、一岗双责、齐抓共管、失职追责”的安全生产责任制，加强重点行业和人员密集场所的安全监管，加大隐患排查整治力度，推进安全社区建设，坚决防止重特大事故发生。做好地质灾害防治、防汛抗旱和森林防火工作。实施最严格的食品药品监管，严防食品药品安全事件发生。创新社会治理，完善社会治安防控体系，排查化解社会矛盾纠纷，强化征地拆迁规范化管理。严厉打击违法犯罪活动，加强反恐防暴工作，深入开展毒品、“盗抢骗”专项打击行动，做好流动人口和特殊人群服务管理。深化公安、司法行政体制改革，加快派出所、司法所规范化建设。推进依法治市，持续开展“法律七进”工作。健全协调联动和快速反应机制，加强国防后备力量和应急队伍建设，提高应急处置能力。

　　（八）加强政府自身建设

　　建设法治政府。坚持重大事项向人大报告、规范性文件向人大备案，主动接受人大法律监督、工作监督，认真办理人大代表议案、建议批评意见。坚持合作共事，自觉接受政协民主监督，办好政协提案。广泛听取民主党派、工商联、无党派人士和人民团体的意见。积极支持工会、共青团、妇联、残联等群团组织开展工作。高度重视司法监督、舆论监督，激发群众监督正能量。落实《法治政府建设实施纲要》，配合做好甜城湖保护等立法工作。加强政府智库建设，推进综合执法，深化政务公开，强化行政复议，不断提升政府科学、民主、依法行政水平。

　　建设效能政府。深入学习习近平总书记系列重要讲话精神，全面贯彻落实中央和省委、市委决策部署，切实加强政府系统党的建设。以“敢挺、苦干、实拼”的智慧和力量，改进作风，推进工作，巩固“三严三实”“两学一做”成果。深化“放管服”改革，以清单管理推动简政放权，清理规范行政审批和公共服务事项。推进“互联网+政务服务”，加快建设网上政务大厅，实现“双随机、一公开”监管全覆盖。健全正向激励机制，完善工作责任、督查问责和第三方绩效评价机制，整肃“庸懒散浮拖”行为，增强行政执行力。强化预算管理，提高财政资金使用绩效。
